# Treatment Outcome of Severe Respiratory Type B Tularemia Using Fluoroquinolones

**DOI:** 10.1093/cid/ciad690

**Published:** 2024-01-31

**Authors:** Micael Widerström, Sara Mörtberg, Mattias Magnusson, Peter Fjällström, Anders F Johansson

**Affiliations:** Department of Clinical Microbiology, Umeå University, Umeå, Sweden; Department of Infectious Diseases, Östersund Hospital, Östersund, Sweden; Department of Radiology, Östersund Hospital, Östersund, Sweden; Department of Clinical Microbiology, Umeå University, Umeå, Sweden; Department of Clinical Microbiology, Umeå University, Umeå, Sweden

**Keywords:** fluoroquinolone/therapeutic use, treatment outcome, tularemia/drug therapy, computed tomography, X ray

## Abstract

**Background:**

Fluoroquinolones lack approval for treatment of tularemia but have been used extensively for milder illness. Here, we evaluated fluoroquinolones for severe illness.

**Methods:**

In an observational study, we identified case-patients with respiratory tularemia from July to November 2010 in Jämtland County, Sweden. We defined severe tularemia by hospitalization for >24 hours and severe bacteremic tularemia by *Francisella tularensis* subsp. *holarctica* growth in blood or pleural fluid. Clinical data and drug dosing were retrieved from electronic medical records. Chest images were reexamined. We used Kaplan–Meier curves to evaluate time to defervescence and hospital discharge.

**Results:**

Among 67 case-patients (median age, 66 years; 81% males) 30-day mortality was 1.5% (1 of 67). Among 33 hospitalized persons (median age, 71 years; 82% males), 23 had nonbacteremic and 10 had bacteremic severe tularemia. Subpleural round consolidations, mediastinal lymphadenopathy, and unilateral pleural fluid were common on chest computed tomography. Among 29 hospitalized persons with complete outcome data, ciprofloxacin/levofloxacin (n = 12), ciprofloxacin/levofloxacin combinations with doxycycline and/or gentamicin (n = 11), or doxycycline as the single drug (n = 6) was used for treatment. One disease relapse occurred with doxycycline treatment. Treatment responses were rapid, with median fever duration 41.0 hours in nonbacteremic and 115.0 hours in bacteremic tularemia. Increased age-adjusted Charlson comorbidity index predicted severe bacteremic tularemia (odds ratio, 2.7 per score-point; 95% confidence interval, 1.35–5.41). A 78-year-old male with comorbidities and delayed ciprofloxacin/gentamicin treatment died.

**Conclusions:**

Fluoroquinolone treatment is effective for severe tularemia. Subpleural round consolidations and mediastinal lymphadenopathy were typical findings on computed tomography among case-patients in this study.

## Teaser

In an observational study of severe respiratory tularemia acquired by inhalation of *Francisella tularensis* aerosols, ciprofloxacin and levofloxacin appeared to be highly effective for treatment. Results were consistent between bacteremic and nonbacteremic illness.

Inhalation of the bacterium *Francisella tularensis* in aerosols can result in clinical presentation of respiratory or pneumonic tularemia, which may be a severe infection [1]. Inhalation transmission occurs rarely in natural outbreaks but is considered the most likely if *F. tularensis*, which is listed as a Tier 1 agent, would be deliberately released. There are 2 *F. tularensis* subspecies of primary clinical relevance, *F. tularensis* subsp*. tularensis* (Jellison type A) and *F. tularensis* subsp*. holarctica* (Jellison type B) [2, 3]. Disease presentation and pathophysiology are similar, but type A tularemia in humans has been exclusively documented on the North American subcontinent and is generally considered more aggressive with higher mortality [1]. More recent research using genetic typing has identified heterogeneity in mortality risks among type A genetic varieties and found that type B varieties may cause risk similar to that caused by some type A varieties. Moreover, the role of patient-associated factors in relation to severity of disease remains unclear [4, 5].

Current antibiotic treatment guidelines for respiratory tularemia rely on limited and mostly anecdotal clinical experience found in the literature. Consequently, updated guidelines need to be developed. Streptomycin has been considered the drug of choice for all clinical forms of severe tularemia; however, its use has substantially decreased in recent decades due to side effects including vestibular toxicity [1, 3]. Gentamicin and other aminoglycosides may substitute for streptomycin, but treatment efficacy may be lower [1, 3, 6–8]. Other treatment alternatives include tetracycline, doxycycline, and chloramphenicol, which are bacteriostatic agents associated with risk of disease relapse and traditionally not preferred in severe tularemia [1, 6, 9]. Tetracyclines are a valuable option in less severe disease and for prophylaxis of tularemia acquired by inhalation [10, 11]. Fluoroquinolones lack US Food and Drug Administration and European Medical Agency approval for tularemia but have been extensively used for treatment of patients with less severe illness [12–17]. There are limited data on treatment outcomes with fluoroquinolones in respiratory tularemia; a handful of cases have been described from the United States, and a previous epidemiological study from Finland suggests that these drugs may be valuable options [18, 19].

To assess outcomes with fluoroquinolone treatment for severe tularemia, we conducted a single-center, retrospective, observational study of patients who acquired tularemia by inhalation during a local outbreak in Sweden in 2010 [20].

## METHODS

### Case Definition and Identification

Case-patients were identified by the County Medical Office in Jämtland County, Sweden, in SmiNet, the electronic reporting system for notifiable diseases. The case definition required laboratory verification and proof of respiratory tularemia by chest imaging and/or a clear clinical description of an acute lower respiratory tract infection on initial presentation. Laboratory verification required growth of *F. tularensis* in blood or pleural fluid cultures and/or a 4-fold increase in *F. tularensis*-specific antibody titers (immunoglobulin M and/or immunoglobulin G) between 2 blood samples. Severe tularemia was defined by the need for hospital care for at least 24 hours.

### Laboratory Diagnostics

Laboratory studies were performed as part of the clinical evaluation for the persons identified as case-inpatients or case-outpatients and included biochemistry and clinical microbiology workup. With 1 exception, at least 1 blood culture set was taken for all case-inpatients before the start of any appropriate antimicrobial drug treatment against *F. tularensis.* The exception was a person who had 2 oral doses of ciprofloxacin as an outpatient before being admitted to hospital where blood culture was performed. One blood culture set consisted of 2 aerobic and 2 anaerobic bottles (BACTEC Plus blood culture system; Becton Dickinson Diagnostic Instrument Systems, Sparks, MD). For 1 case-inpatient, pleural fluid samples were cultured. *Francisella tularensis* diagnostics included polymerase chain reaction (PCR)-based direct detection in clinical specimens and agglutination test for species identification of cultures as previously described [3, 20].

### Study Design and Clinical Data Variables

Electronic medical records were reviewed to collect data including clinical progression of disease, vital signs, body temperature, biochemical test results, and clinical microbiology results. Underlying medical conditions were determined by complete *International Classification of Diseases, Tenth Revision, Clinical Modification* (ICD-10), codes 48 months before the acute tularemia episode. We used an adaptation of Charlson index scoring developed for register-based research in Sweden and the website MDCalc for obtaining age-adjusted Charlson comorbidity indices [21–23]. Disease onset and exposure risk factors for acquiring tularemia were collected via structured telephone interviews by personnel at the County Medical Office in an outbreak investigation. Supplementary Figure 1 shows key elements of the study design (population characteristics, data variables, and key outcomes).

### Antibiotic Treatment Variable

The electronic time stamp of the first dose and the time stamp of the last dose of different antibiotic courses defined treatment durations in hours. If only 1 dose of a drug was given, we used the standard interval between doses as the treatment duration (eg, 1 dose of ciprofloxacin was translated to a 12-hour treatment duration). A single dose of gentamicin was counted as a 24-hour treatment duration. The following appropriate tularemia antibiotics were used for case-inpatients and analyzed: ciprofloxacin, levofloxacin, doxycycline, and gentamicin. Inappropriate antibiotics used and analyzed were of the beta-lactam class (amoxicillin, benzylpenicillin, cefotaxime, ceftriaxone, cefuroxime, cephalexin, meropenem, piperacillin-tazobactam, and phenoxymethylpenicillin).

### Chest Imaging Evaluation

An experienced radiologist (M. M.) retrospectively reevaluated all chest radiographs and computed tomography scans of the thorax of the 67 tularemia case-patients. The interpretation was merged with the original imaging reports.

### Outcomes

Among inpatient cases with severe tularemia, we assessed 3 outcomes: body temperature with defervescence defined as <38°C sustained for at least 72 hours or 2 measurements followed by discharge, time to discharge from the hospital, and 30-day mortality after disease onset.

### Data Processing, Statistical Analyses, and Handling of Missing Data

To analyze the effect of antibiotic treatments, we used Kaplan–Meier curves with log-rank testing of the null hypothesis that different antibiotic treatment groups had identical hazard functions. Confidence intervals were estimated using bias-corrected bootstrapping with 2000 iterations. For 2-group comparison of appropriate and inappropriate antibiotic treatments, we assigned persons who received at least 1 dose of an appropriate antibiotic to the appropriate treatment group. The first appropriate antibiotic dose defined the start. Time, in hours, was counted until the event of interest (defervescence or discharge from the hospital). We assigned persons treated with any of the beta-lactam antibiotics to the inappropriate treatment group. To compare the effects of different appropriate antibiotic treatments, we created 3 groups: case-inpatients who received fluoroquinolones (levofloxacin, ciprofloxacin, or a combination of the 2), those who received doxycycline alone, and those who received fluoroquinolones combined with gentamicin and/or doxycycline. We excluded 4 case-inpatients in the survival analyses due to ambiguous outcome data as follows: 2 inpatients who were discharged and given appropriate antibiotic treatment the same day lacked outcome data, 1 inpatient who had no fever or antibiotics was retrospectively diagnosed after extensive medical in-hospital investigations for chest lymphadenopathy, and 1 inpatient who received a single dose of gentamicin who defervescenced from this treatment or spontaneously and stayed in the hospital for 20 days due to acute renal failure. One patient who died was censored in the survival analysis at the time of death. To estimate the probability of bacteremic versus nonbacteremic severe tularemia among case-inpatients, we used binominal regression based on the age-adjusted Charlson comorbidity index and the age in years. To examine relative risk of bacteremic severe tularemia in relation to ICD-10 diagnostic categories, we used the *χ*^2^ test and data for all case-patients. We used the proportion test for frequencies between patient groups and the *t* test for continuous values. Summary statistics were performed univariately. Data processing was carried out using R version 4.1.2 and RStudio version 1.3.1717 with the R-packages readr (version 2.1.1), tidyverse (version 1.3.1), lubridate (version 1.8.0), xlsx (version 0.6.5), survival (version 3.2–13), survminer (version 0.4.9), mgcv (version 1.8–38), boot (version 1.3–28), and car (version 3.0–13).

### Ethics Committee Approval

The Umeå University Regional Ethical Review Board approved the study.

## RESULTS

### Outbreak Description

Between 16 July 2010 and 27 October 2010, 67 case-patients presented with the respiratory form of tularemia (54 men and 13 women; median age, 66 years; range, 24–90) in Jämtland County, Sweden. All had pathology on chest imaging and/or a clear description of an acute lower respiratory tract infection in the patient record on initial presentation. Probable exposure to aerosolized *F. tularensis* was reported by 94% (63 of 67). Suspected infectious exposures or risk activities included self-reported physical proximity to voles or contact with vole urine or feces (n = 27), performing brush cutting or grass trimming (n = 18), handling wood (n = 9), and walking or moose hunting in the forest (n = 9).

### Clinical Characteristics, Laboratory Verification, and Underlying Medical Conditions

Table 1 shows age, sex, smoking history, comorbidity score, and symptoms of acute illness for case-patients. The proportion for male sex was approximately 80% among both persons clinically managed as inpatients and managed as outpatients. Older age and higher average age-adjusted Charlson comorbidity index were significant predictors for case-inpatients. Gastrointestinal symptoms were more common for case-inpatients. Twenty-three of the 33 inpatients were laboratory verified by a 4-fold increase in *F. tularensis*-specific antibodies between 2 blood samples and are hereafter referred to as having severe tularemia. Ten case-inpatients had at least 1 positive microbial culture for *F. tularensis* in normally sterile specimen types, signifying invasive disease (9 by blood cultures and 1 by pleural fluid culture). For brevity, these are hereafter referred to as having severe bacteremic tularemia. All 34 case-outpatients were laboratory verified by a 4-fold increase in *F. tularensis*-specific antibodies between 2 blood samples.

**Table 1.** Demographic and Clinical Characteristics of Case-Inpatients and Case-Outpatients

Characteristic	Case-Inpatients, n = 33	Case-Outpatients, n = 34	*P* Value^a^
Median age (range), y	71 (47–90)	60 (24–90)	.0035
No. male (%)	27 (82)	27 (79)	1.00
No. smoking (%)	3 (9)	2 (6)	.97
Mean age-adjusted Charlson comorbidity index (range)	3.78 (0–10)	1.74 (0–4)	<.0001
No. myalgia (%)	26 (79)	23 (68)	.41
No. cough (%)	20 (61)	25 (74)	.31
No. headache (%)	16 (48)	9 (26)	.080
No. nausea (%)	9 (27)	2 (6)	.023
No. vomiting (%)	6 (18)	0 (0)	.011
No. diarrhea (%)	6 (18)	0 (0)	.011

^a^Two-tailed *t* test was used for continuous values. The proportion test was used for frequencies.

An impaired level of consciousness at admission was more common for persons with severe bacteremic tularemia (5 of 10) than for persons with severe nonbacteremic tularemia (1 of 23) (*P* = .0054). There was no significant difference at admission in median heart rate between persons being case-inpatients with or without *F. tularensis* bacteremia (80 bpm in both groups), in median systolic blood pressure (124 and 140 mmHg), respiratory rate (20 and 19), or median temperature (39.2°C and 39.2°C). The median duration of hospitalization was 13 days (range, 7–23) for individuals with severe bacteremic tularemia and 3 days (range, 1–10) for individuals with nonbacteremic tularemia. In binominal regression analysis, the age-adjusted Charlson comorbidity index was associated with severe bacteremic tularemia (n = 33; odds ratio, 2.7 per score-point; 95% confidence interval [CI]: 1.35–5.41), suggesting that patient factors were important for more invasive tularemia. The variable age, in years, was nonsignificant in the analysis. Among different ICD-10 diagnostic categories, congestive heart failure was significantly associated with bacteremic tularemia (N = 67; relative risk, 4.5; 95% CI: 1.9–10.7).

### Chest Imaging

**Figure 1.**
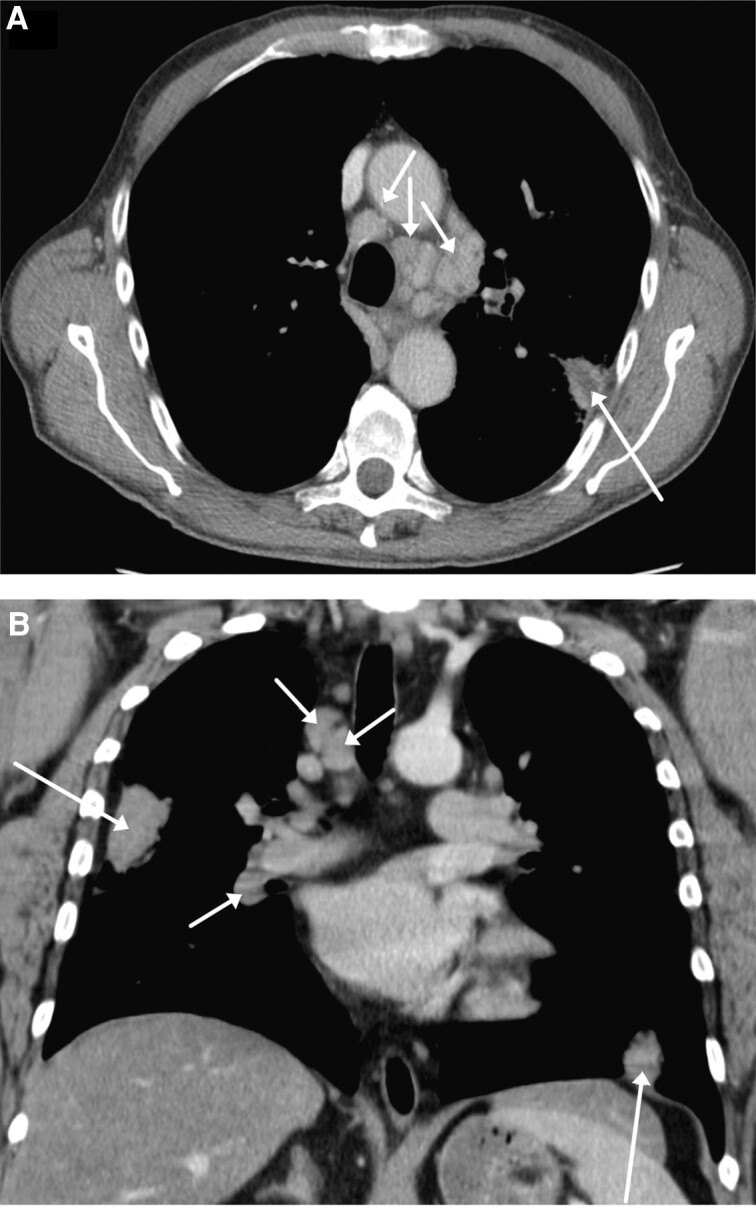
*A,* Axial computed tomography section of the thorax of a 68-year-old male case-patient 10 days after disease onset. A 2.5-cm subpleural consolidation with central necrosis is indicated by the long arrow. Mediastinal reactive lymph nodes are indicated by short arrows. *B,* Coronal computed tomography section of the thorax of a 48-year-old male case-patient 7 days after disease onset. Over the previous 24 hours, he developed a dry cough. Bilateral subpleural consolidations are indicated by long arrows. Moderately enlarged paratracheal lymph nodes are indicated by short arrows. Image credit: Region Jämtland-Härjedalen.

Table 2 summarizes chest imaging results for the 67 case-patients. Imaging pathology was detected in 82% (41 of 50) of chest radiographs and all (24 of 24) computed tomography images. Consolidations in the lung parenchyma, pleural fluid, and/or enlarged lymph nodes were common findings (Figure 1). Rounded consolidations of 1–3 cm, predominantly in the subpleural region with a diffuse marginal zone, were typical on computed tomography, sometimes with signs of inclusion necrosis. Modest lymph node enlargement in the mediastinal and hilar regions with a short-axis width measuring 1–1.5 cm, sometimes with necrotic inclusions, was very common on computed tomography. Unilateral small to moderate amounts of pleural fluid were common, especially for case-inpatients. Characteristic chest images are compiled in Supplementary Figures 2–5.

**Table 2.** Chest Imaging Findings

Finding	Case-Inpatients		Case-Outpatients	
	Radiography, n = 32	Computed Tomography, n = 18	Radiography, n = 18	Computed Tomography, n = 6
Any abnormality (%)	29 (91)	18 (100)	12 (67)	6 (100)
Presence of mediastinal widening/lymphadenopathy (%)	10 (31)	17 (94)	3 (17)	6 (100)
Presence of pleural effusion (%)	13 (41)	11 (61)	3 (17)	1 (17)
Median amount of pleural effusion, (range), mm^a^		17.5 (5–70)		9 (9–9)
Presence of infiltrates/consolidation (%)	26 (81)	15 (83)	10 (56)	4 (67)
No. of lobes involved, median (range)^b^	1 (1–4)	1 (1–5)	1 (1–3)	1.5 (1–3)

^a^Applicable for computed tomography of the thorax.

^b^Applicable for case-patients with infiltrates/consolidation.

### Antibiotic Treatment and Outcomes

A 78-year-old case-inpatient male with an age-adjusted Charlson comorbidity index score of 7 died on day 23 after disease onset. He was admitted 3 days after disease onset and initially received inappropriate antibiotic treatment before initiation of ciprofloxacin on day 11. The clinical course indicated sepsis with organ failure. Autopsy indicated acute circulatory failure with acute myocardial infarction, infarction of the small intestine, and edema of the lungs. Tracheal and bronchial mucosa were mildly inflamed. A biopsy from a paratracheal lymph node showed necrotic lesions, but PCR and stainings for microscopy were *F. tularensis*-negative. All other case-patients survived 30 days, translating to 30-day mortality of 1.5% (1 of 67) in the full cohort.

**Figure 2.**
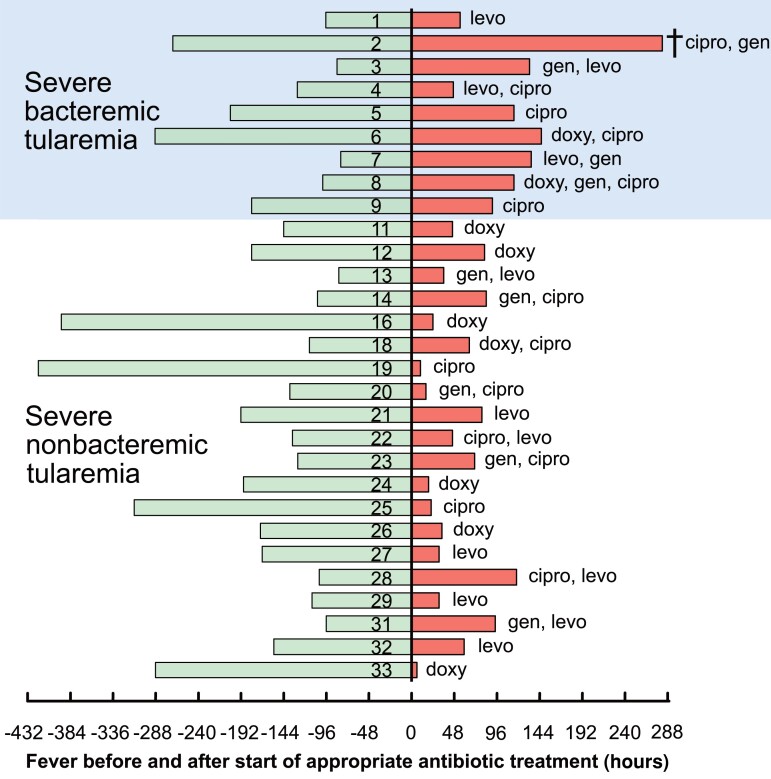
Fever duration including a self-reported period at home before the start of appropriate antibiotic treatment against *Francisella tularensis* is shown as bars to the left. Numbers 1–33 at the center identify individual case-inpatients. Defervescence and the respective appropriate antibiotics administered are shown as bars to the right. A cross indicates that 1 person died. Abbreviations: cipro, ciprofloxacin; doxy, doxycycline; gen, gentamicin; levo, levofloxacin.

Hereafter, outcomes for the 29 case-inpatients with complete treatment and outcome data are described in relation to antibiotic treatment including the person who died. Overall, delays in appropriate antibiotic treatment (ciprofloxacin, levofloxacin, gentamicin, or doxycycline) were common, with a median duration of fever 149.5 hours prior to appropriate antibiotic treatment (n = 20; 82.0–420.0) in severe nonbacteremic tularemia and 128.5 hours (n = 9; range, 84.0–288.5) in severe bacteremic tularemia. Median duration of fever after the initiation of appropriate antibiotic treatment was 41.0 hours (n = 20; 6.0–118.0) in severe nonbacteremic tularemia and 115.0 hours (n = 9; range, 47.0–282.0) in severe bacteremic tularemia (Figure 2).

**Figure 3.**
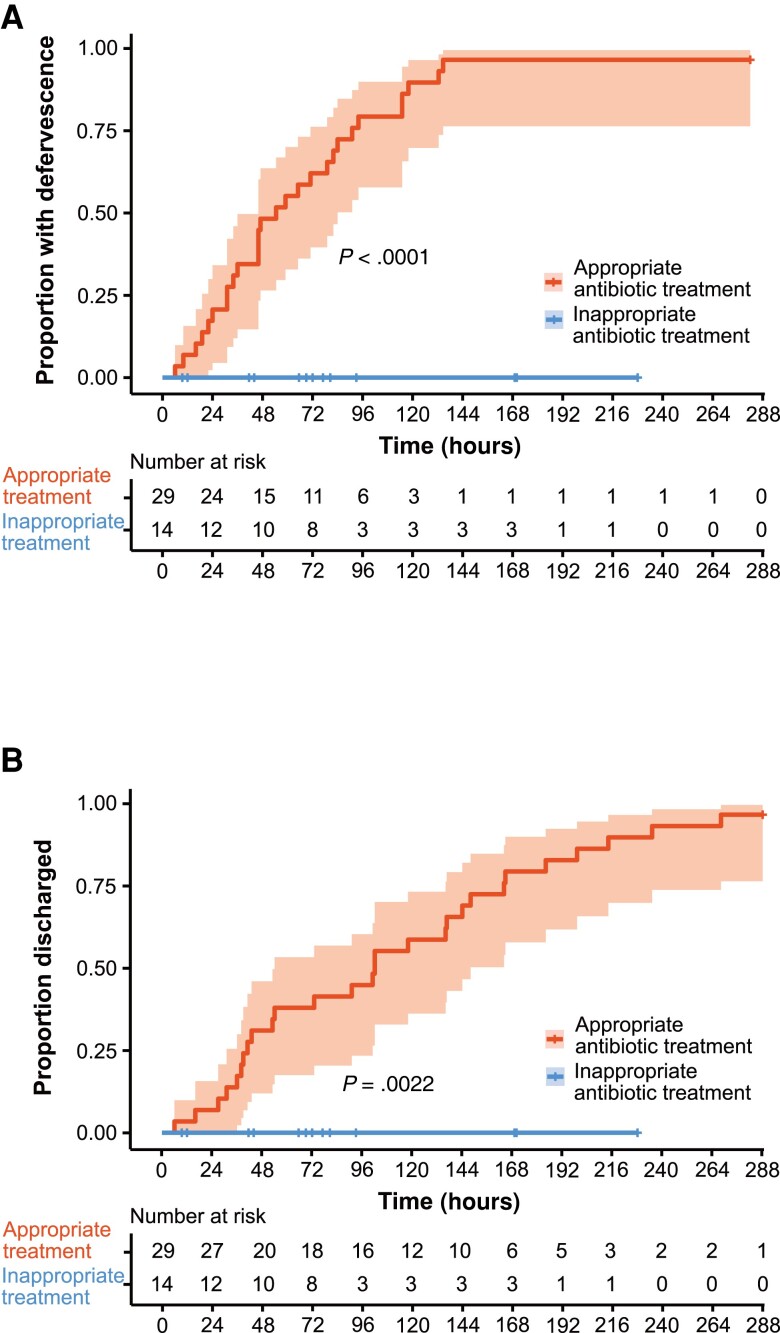
Kaplan–Meier curves of treatment outcomes. The time to defervescence (*A*) and discharge from the hospital (*B*) are shown. A red line indicates appropriate antibiotic treatment for tularemia and a blue line indicates inappropriate antibiotic treatment for tularemia. In total, 29 case-inpatients were evaluated, with 14 initially receiving inappropriate antibiotics. The 95% confidence intervals are shown by shading.

Survival analysis of time to defervescence or discharge from the hospital comparing appropriate and inappropriate antibiotic treatment showed a clear difference in the outcomes (Figure 3). In the inappropriate treatment group, none defervesced and none were discharged. In contrast, after receiving antibiotics with antimicrobial activity against *F. tularensis*, case-inpatients defervesced (Figure 3*A*) and were discharged (Figure 3*B*).

**Figure 4.**
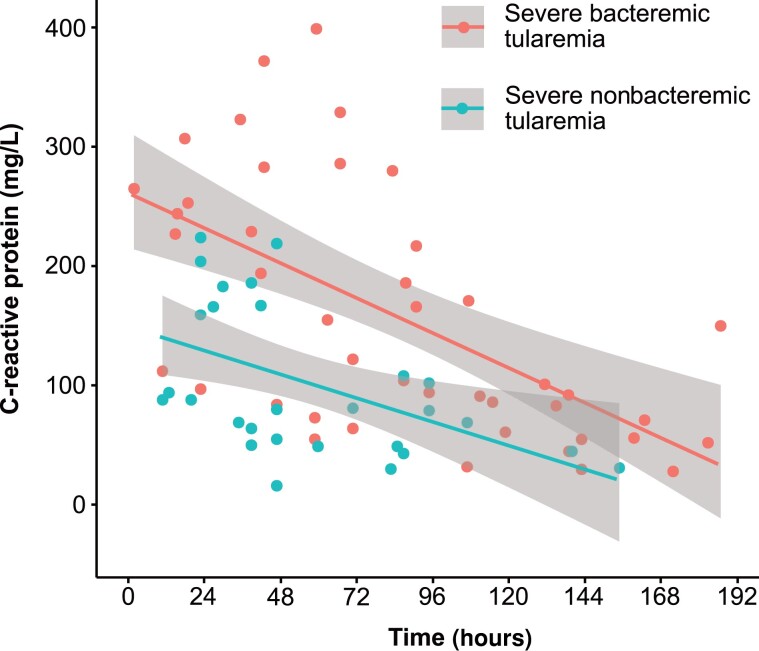
Dynamics over time of C-reactive protein levels in blood. Time 0 at the *x*-axis indicates the initiation of at least 1 appropriate antibiotic. The data represent 9 case-inpatients with severe bacteremic tularemia and 20 case-inpatients with nonbacteremic severe tularemia with complete treatment outcome data. The 95% confidence intervals are shown by shading.

Analysis of C-reactive protein levels in blood after the initiation of appropriate antibiotic treatment reflected the favorable treatment outcomes and showed that bacteremia was associated with a stronger inflammatory response (Figure 4).

**Figure 5.**
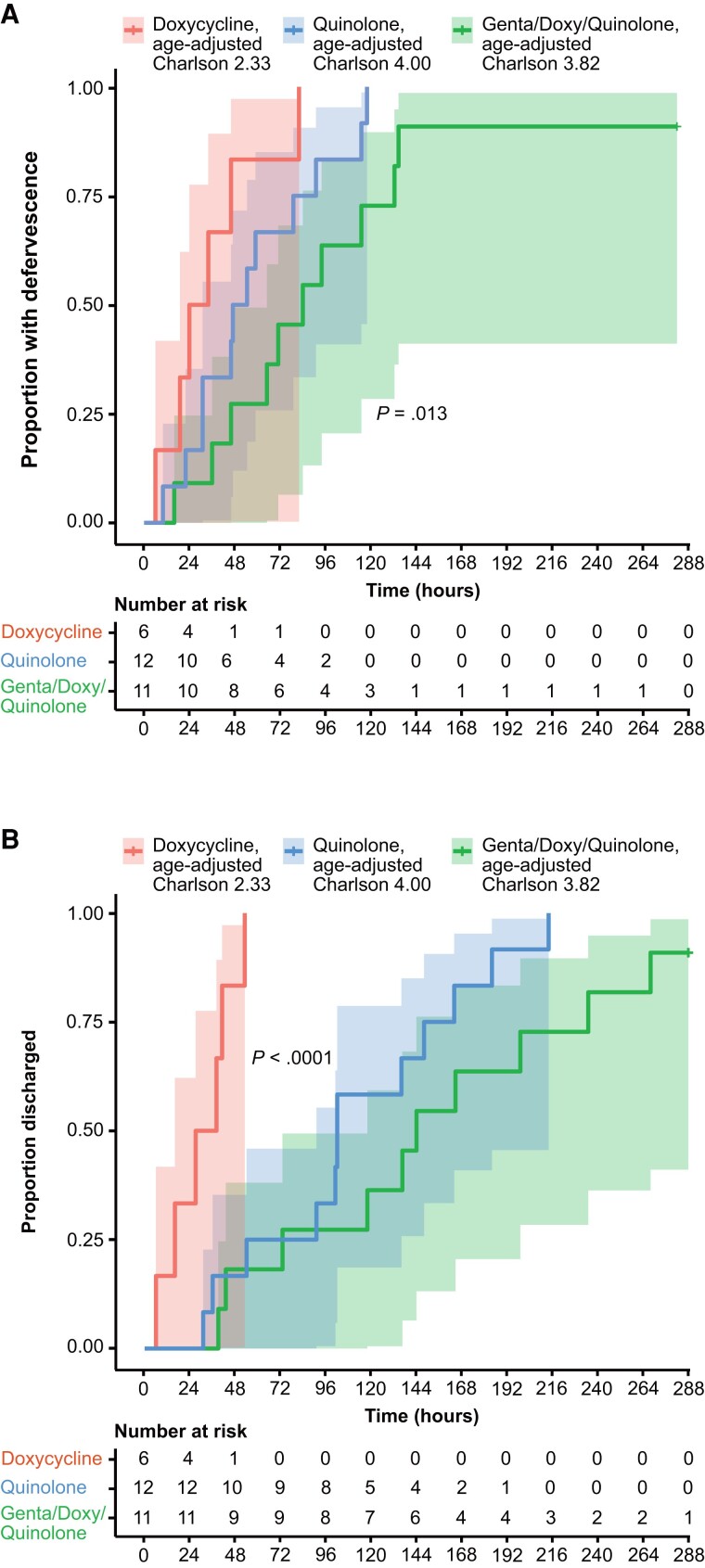
Kaplan–Meier curves of treatment outcome divided by 3 groups of appropriate antibiotic treatments for *Francisella tularensis*. Defervescence (*A*) and discharge from the hospital (*B*) are shown. A red line indicates doxycycline, a blue line indicates ciprofloxacin and/or levofloxacin, and a green line indicates combinations of ciprofloxacin and/or levofloxacin with gentamicin or doxycycline. Mean age-adjusted Charlson comorbidity indices are shown at the color labels indicating the 3 treatment groups. The 95% confidence intervals are shown by shading. Abbreviations: doxy, doxycycline; genta, gentamicin.

The order of different appropriate antibiotic courses given and days of therapy per person among case-inpatients are given in Supplementary Table 1, illustrating that fluoroquinolones were a mainstay of treatment. A cumulative quantification of all appropriate antibiotics added up to 2022.5 treatment hours until defervescence in the cohort of 29 case-inpatients: 73% fluoroquinolones (761.0 ciprofloxacin and 703.5 levofloxacin hours), 10% gentamicin (208.0 hours), and 17% doxycycline (350.0 hours). A prescription bias with regard to patient factors was suggested by analysis of age-adjusted Charlson comorbidity indices; case-inpatients who received doxycycline as a single drug had an average score of 2.33 (n = 6; range, 1–4), while persons who received fluoroquinolones only or fluoroquinolones combined with gentamicin and/or doxycycline had average scores of 4.00 (n = 12; range, 0–7) and 3.82 (n = 11; range, 0–7), respectively. Overall, the time to defervescence patterns indicated rapid responses for all 3 comparator groups considering the severe infection, with similar but a more extended pattern for time to discharge (Figure 5).

One of the 29 persons had a disease relapse after a 15-day course of doxycycline as the single drug. Fever recurred 19 days after discharge for this person but rapidly diminished with a secondary treatment using oral ciprofloxacin (Supplementary Table 1).

**Figure 6.**
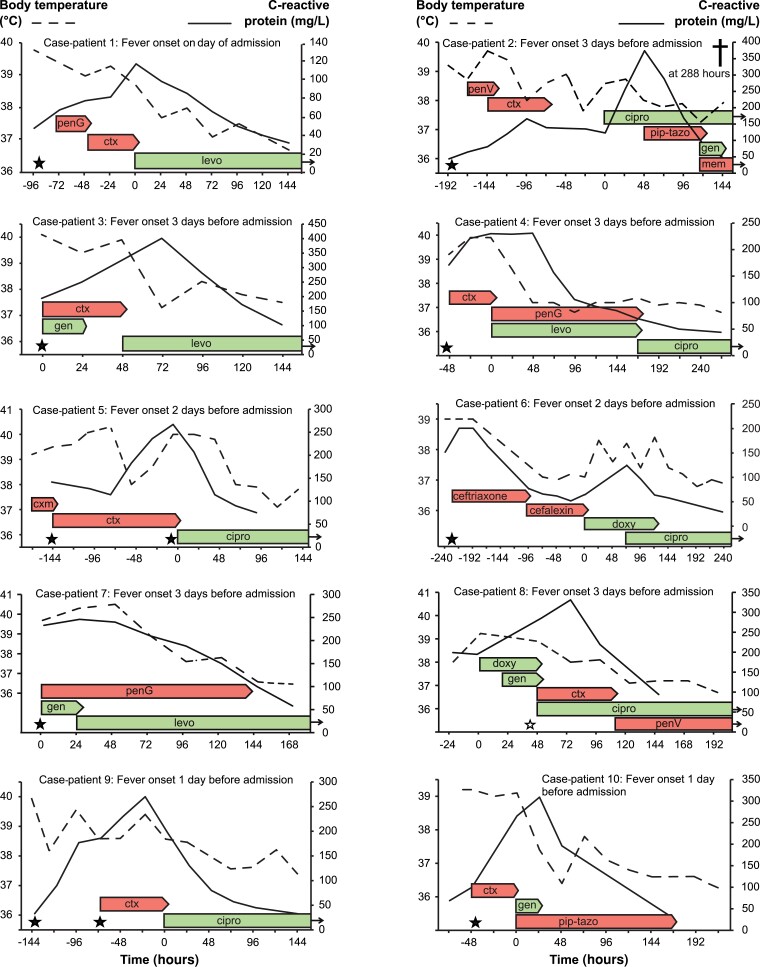
Fever and C-reactive protein dynamics after admission to the hospital in relation to appropriate antibiotic treatment (green bar) and inappropriate antibiotic treatment (red bar) among 10 persons with severe bacteremic tularemia. A black star indicates the time point for collecting a positive blood culture. An open star indicates the time point for collecting a positive pleural fluid culture. A cross indicates that 1 person died. Data were truncated after defervescence. Continued antibiotic administration is indicated by a small black arrow to the far right. Abbreviations: cipro, ciprofloxacin; ctx, cefotaxime; cxm, cefuroxime; doxy, doxycycline; gen, gentamicin; levo, levofloxacin; mem, meropenem; penG, penicillin G; penV, penicillin V; pip-tazo, piperacillin-tazobactam.

Individual characteristics of body temperature and C-reactive protein responses among the 10 persons with severe bacteremic tularemia are provided in Figure 6. The tenth person who was excluded from formal survival analyses of treatment outcomes due to ambiguous outcome data had preexisting mild renal impairment and empirically received a single dose of gentamicin. Clinical septic shock with acute renal failure occurred with creatinine levels 400–500 mg/L followed by a hospital stay of 20 days.

## DISCUSSION

In this unusually large natural outbreak of respiratory type B tularemia, fluoroquinolone antibiotics were useful for treatment of severe disease. The results were consistent between bacteremic and nonbacteremic severe disease. We found that disease severity was dependent on age and comorbidities, emphasizing that patient factors are highly relevant when selecting antibiotic treatment. We found that clinicians selected appropriate antibiotics guided by their judgment of patient factors, disease severity, and their understanding of drug effects, with fluoroquinolones being a mainstay of treatment. In the single fatal outcome of the outbreak, patient factors including comorbidities and age likely contributed.

Typical clinical presentation was onset of an influenza-like illness followed by dry cough that frequently occurred >7 days after fever onset, which is consistent with previous knowledge of tularemia acquired by inhalation in humans and in experimental animal models [7, 18, 24–26]. Our data are consistent with previous reported challenges in recognizing inhalational tularemia by the symptoms classically associated with pneumonia. We concur with prior views that historically there has been a frequent misclassification into the entity “typhoidal tularemia” [27]. We advocate for using the terms “respiratory tularemia” or “pneumonic tularemia” to describe this form of tularemia, which can manifest as a severe infection that meets sepsis criteria [27, 28]. We show that chest imaging and, in particular, computed tomography reveal findings that are typical for respiratory tularemia and can be used to identify the clinical form of the disease. The imaging findings described here in humans correlate well with the lung pathology observed in non-human primates experimentally infected by aerosolized *F. tularensis* having raised necrotizing and/or pyogranulomatous foci on lung lobes at autopsy [24, 29]. We found that rounded consolidations in the subpleural region with a diffuse marginal zone and sometimes with signs of inclusion necrosis were common findings in humans. Other findings were mediastinal lymph node enlargement, sometimes with necrotic inclusions and often accompanied by unilateral pleural fluid, again consistent with pathology in experimentally infected non-human primates.

There were significant delays in diagnosis and time to initiation of antibiotics. The presence of initially mild respiratory symptoms and subtle abnormalities on standard chest X-rays contributed to the delays. Considering the delays, the observed treatment responses were surprisingly rapid. The survival analyses of treatment outcomes confirm that beta-lactam antibiotics are clinically ineffective. Conversely, ciprofloxacin, levofloxacin, doxycycline, and gentamicin coincided with positive treatment outcomes, typically within 1–10 days. The fluoroquinolones levofloxacin and ciprofloxacin were the drugs most frequently used and were effective for severe illness among case-inpatients. We observed that clinicians selected these drugs for more severe illness, sometimes together with gentamicin and/or doxycycline, which is in line with experience from Finland [18].

Our study has several limitations. First, due to its observational design, the choice of antibiotic treatment was nonrandom, for example, it was evident that clinicians selected doxycycline for patients who they judged to be at lower risk. Second, the natural history of tularemia may include spontaneous resolution of disease, and we did not account for this. Third, we lacked detailed outcome data on some patients and excluded these data, potentially conferring bias. Fourth, and most important, multiple types of confounding including the above factors cannot be accounted for in an observational design, making causal relationship between antibiotics and outcome less reliable.

In conclusion, in this observational study, the fluoroquinolone antibiotics ciprofloxacin and levofloxacin appear to be highly effective for treating severe respiratory type B tularemia.

## Supplementary Data

Supplementary materials are available at *Clinical Infectious Diseases* online. Consisting of data provided by the authors to benefit the reader, the posted materials are not copyedited and are the sole responsibility of the authors, so questions or comments should be addressed to the corresponding author.

## Supplementary Material

ciad690_Supplementary_Data

## Notes

***Author Contributions.*** A. F. J. conceived of the study. M. W. and S. M. carried out and compiled results from the outbreak investigation. M. W. collected clinical data and performed analyses. M. M. reevaluated all chest images. P. F. and A. J. designed the statistical analyses. P. F. organized the data and performed the statistical analyses. M. W. and A. F. J. wrote the draft. All authors reviewed and approved the manuscript.

***Acknowledgments.*** We thank the patients and clinically responsible physicians who collected epidemiological information during the outbreak and Joel Terriques and Christina Nelson for their valuable input on the manuscript text.

***Supplement sponsorship.*** This article appears as part of the supplement “Tularemia: Update on Treatment and Clinical Findings,” sponsored by the Centers for Disease Control and Prevention.

***Financial support.*** This work was supported by research grants from the Research and Development Unit, Region Jämtland-Härjedalen and Region Västerbotten.
