# Supplementary material for: Treatment Outcome of Severe Respiratory Type B Tularemia Using Fluoroquinolones
Source: Clin Infect Dis. 2024 Jan 31;78(Suppl 1):S38–46. doi: 10.1093/cid/ciad690 (PMC10828930; doi:10.1093/cid/ciad690)
Supplement: ciad690_Supplementary_Data [file ciad690_supplementary_data.pdf]

SUPPLEMENTARY MATERIALS

Widerström et al. Treatment outcomes of severe respiratory type B tularemia using fluoroquinolones.

Table of contents:

Supplemental Figure S1 - S5 Page 1 - 5

Supplemental Table S1 Page 6

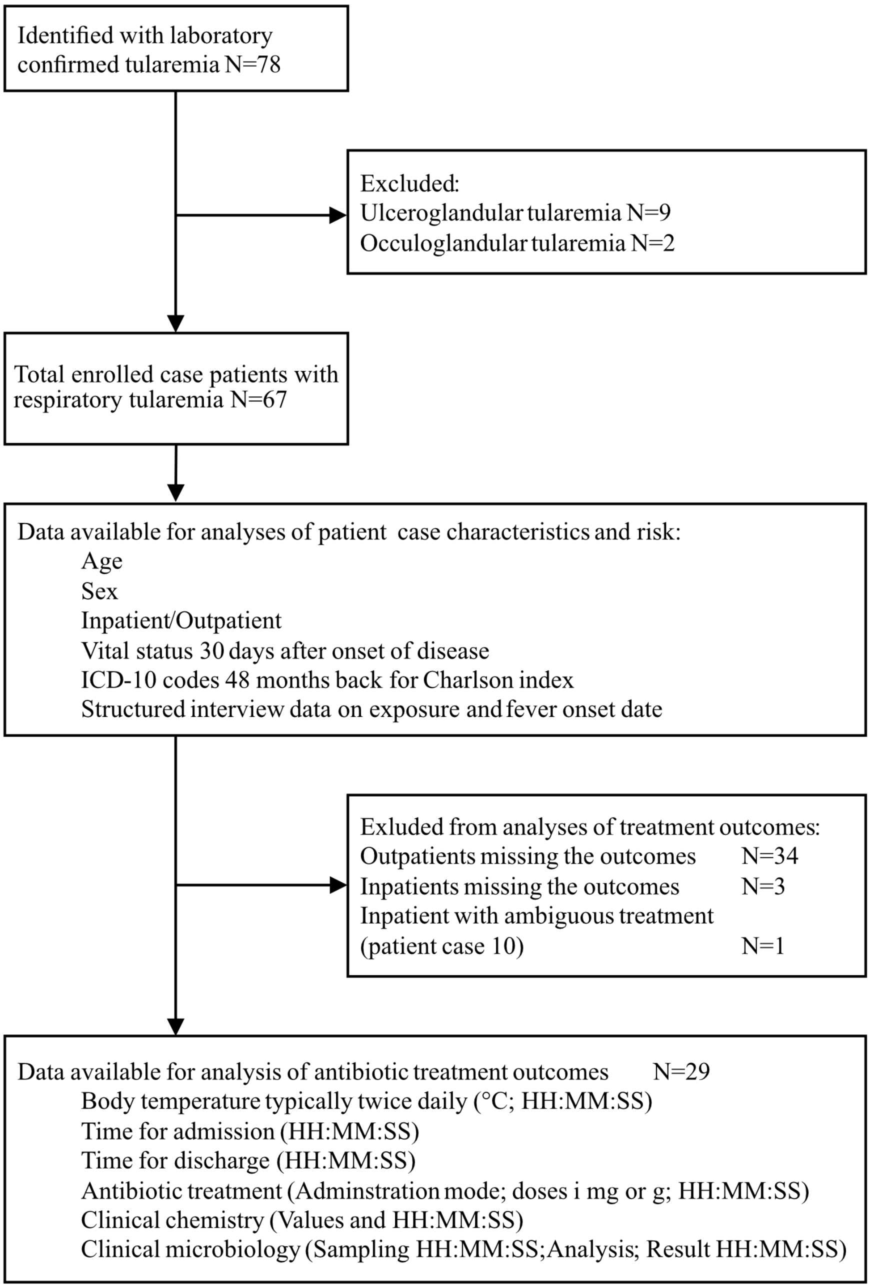

Figure S1. Flow diagram with key elements of the population, data variables, and outcomes.

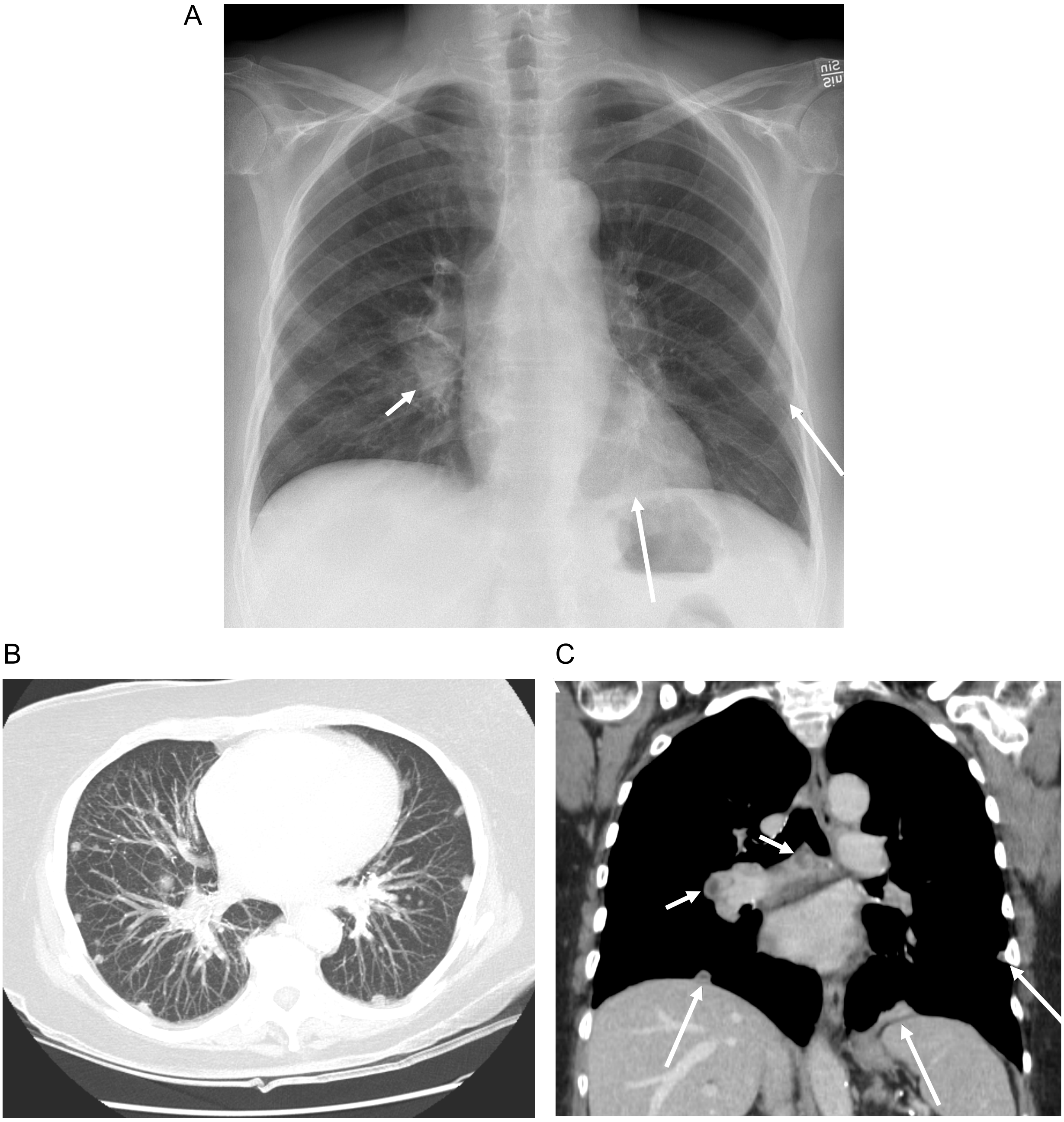

Figure S2. A 72-year-old female case patient with a 5-day history of fever. Frontal projection chest X-ray (A), an axial maximum intensity projection volume CT scan of the thorax (B), and a coronal thorax CT section (C) demonstrate multiple metastasis-like subpleural consolidations with mediastinal and hilar lymphadenopathy. Central necrosis is observed in the lung consolidations and in the pathological lymph nodes. Image credit: Region Jämtland-Härjedalen.

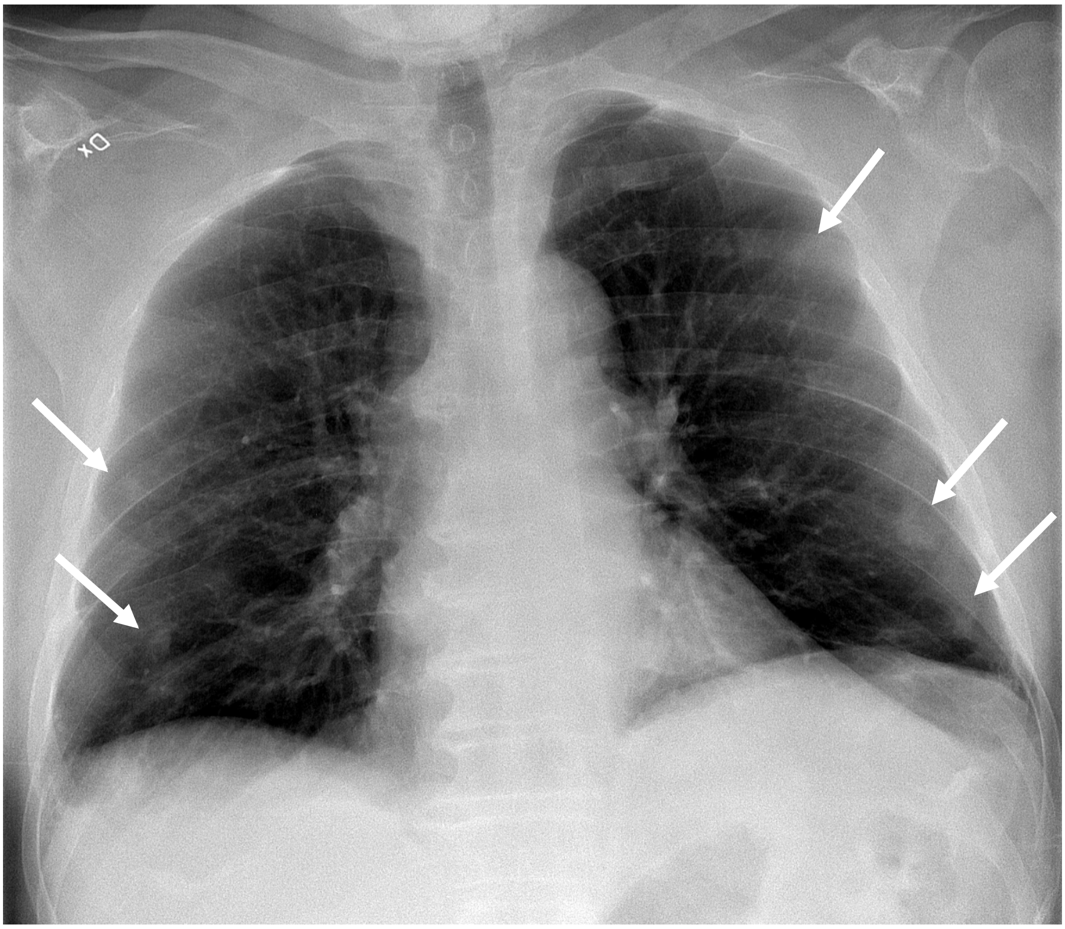

Figure S3. A 77-year-old male case patient with a 7-day history of fever and myalgia. A frontal projection chest X-ray shows scattered, peripheral round consolidations in both lung fields which are uniformly sized at approximately 1 cm. Image credit: Region Jämtland-Härjedalen.

A B

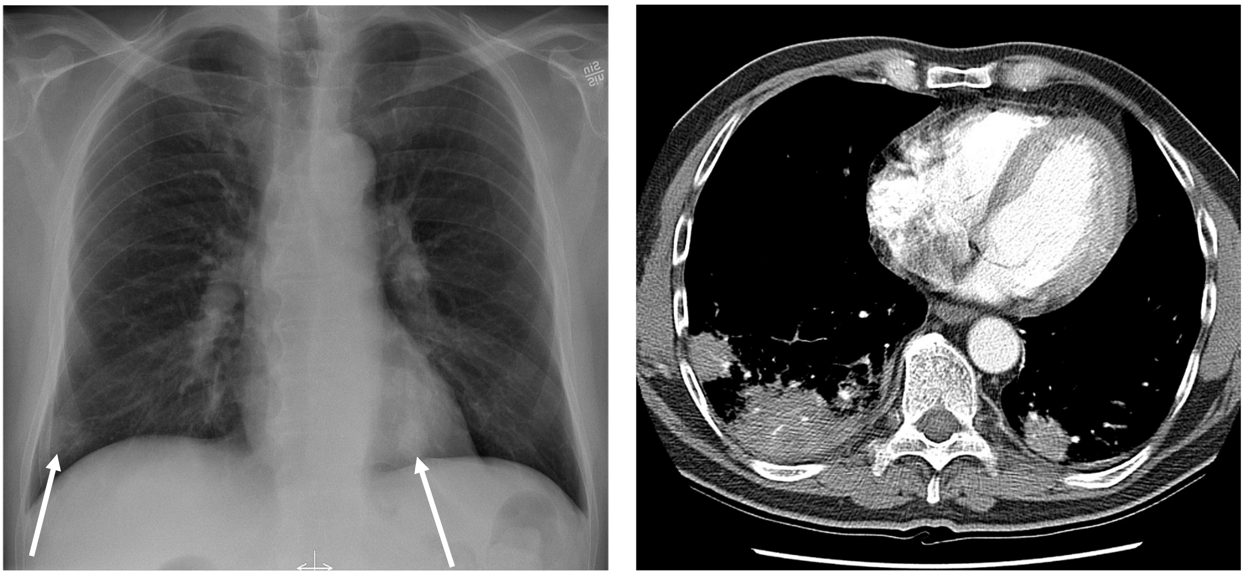

Figure S4. A previously healthy 57-year-old male case patient. A frontal chest X-ray (panel A) at day two after disease onset shows basal rounded consolidations. The patient reported high fever and right-sided chest pain that worsened with respiration. An axial CT thorax section (panel B) from day four after disease onset shows typical rounded consolidations with a basal bilateral distribution and a partial consolidation of the right lower lobe. Note the absence of necrotic features. Image credit: Region Jämtland-Härjedalen.

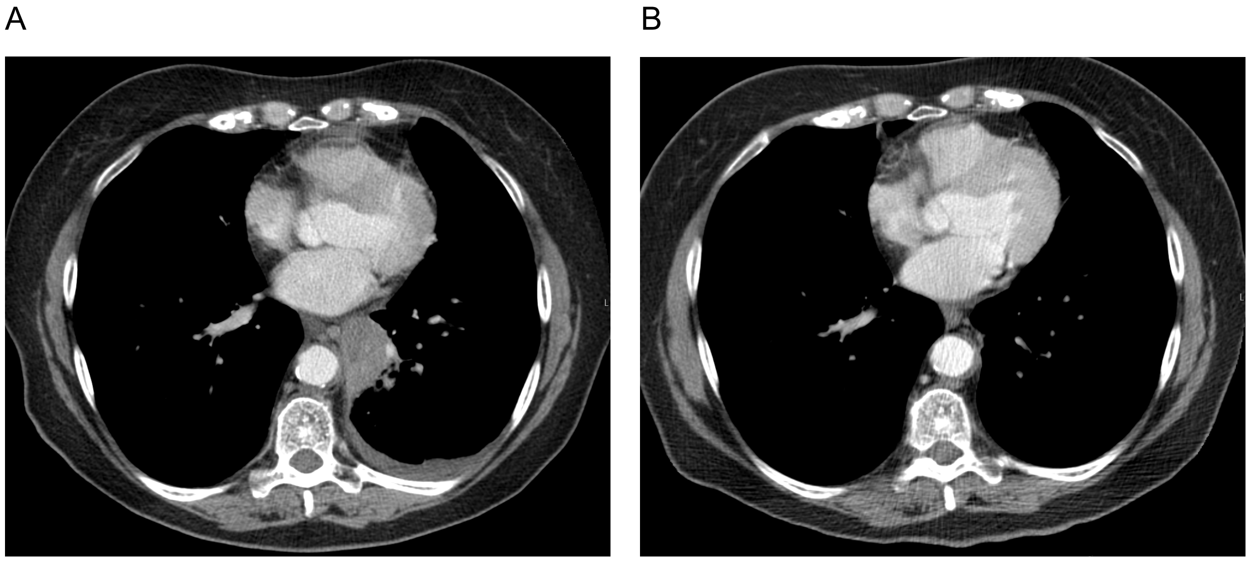

Figure S5. A 70-year-old smoking female case patient presenting with a 14-day history of high fever. An axial CT scan section of the thorax (panel A) reveals a 3.5 cm tumor-like lesion located medially in the left lower lobe and basal pleural fluid on the left side. Transthoracic biopsy confirmed the presence of granuloma consistent with tularemia. A follow-up CT scan (panel B) performed 4.5 months after initiating treatment shows significant regression of the lesion. Image credit: Region Jämtland-Härjedalen.

Table S1. Characteristics of antibiotic treatment among 29 case patients with severe tularemia that were evaluated for the treatment outcomes defervescence and discharge from hospital.

Patient case no.	Fever duration before the start of appropriate antibiotic treatment (Days)	First appropriate antibiotic (Days of therapy)	Second appropriate antibiotic (Days of therapy)	Third appropriate antibiotic (Days of therapy)	Fever relapse (Name of antibiotic with relapse)
1	4.0	levofloxacin (10)			No
2	11.2	ciprofloxacin (12)	gentamicin (1)		NA*
3	3.5	gentamicin (1)	levofloxacin (9)		No
4	5.4	levofloxacin (7)	ciprofloxacin (14)		No
5	8.5	ciprofloxacin (14)			No
6	12.0	doxycycline (5)	ciprofloxacin (14)		No
7	3.3	gentamicin (1)	levofloxacin (9)		No
8	4.2	doxycycline (2)	gentamicin (1)	ciprofloxacin (14)	No
9	7.5	ciprofloxacin (10)			No
11	6.0	doxycycline (15)	ciprofloxacin (10)		Yes** (doxycycline)
12	7.5	doxycycline (14)			No
13	3.4	gentamicin (1)	levofloxacin (9)		No
14	4.4	gentamicin (1)	ciprofloxacin (9)		No
16	16.4	doxycycline (10)			No
18	4.8	doxycycline (2)	ciprofloxacin (13)		No
19	17.5	ciprofloxacin (13)			No
20	5.7	gentamicin (1)	ciprofloxacin (9)		No
21	8.0	levofloxacin (9)			No
22	5.6	ciprofloxacin (1)	levofloxacin (9)		No
23	5.3	gentamicin (1)	ciprofloxacin (8)		No
24	7.9	doxycycline (9)			No
25	13.0	ciprofloxacin (9)			No
26	7.1	doxycycline (14)			No
27	7.0	levofloxacin (6)			No
28	4.3	ciprofloxacin (2)	levofloxacin (6)		No
29	4.7	levofloxacin (13)			No
31	4.0	levofloxacin (9)	gentamicin (1)		No
32	6.5	levofloxacin (9)			No
33	12.0	doxycycline (9)			No

* Not analyzed because the case patent died.

** Relapse of fever and disease symptoms occurred 19 days after discharge from a hospital stay with initial effective treatment using doxycycline as single drug. This case-patient person had reached the primary treatment outcomes evaluated by survival analyses. Initiation of ciprofloxacin as secondary treatment caused rapid and sustained resolution of fever and illness.
